# Millionaire radiology

**DOI:** 10.4103/0971-3026.76068

**Published:** 2011

**Authors:** Indraneel Bandopadhyaya

**Affiliations:** 437, Asiad Village Complex, New Delhi - 110 049, India. E-mail: dr.indraneel@gmail.com

Dear Sir,

“The Business of Radiology”[1] is so full of truth. This is about the Indian medical education fraternity’s worst kept secrets.

Why does MD Radiology cost 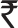 1.5 crore today? What does this truly represent? What next?

**The reasoning:**

1. Knowledge is an asset most sought after in our profession.
2. Anything that is sought after by people has a value. If the commodity is scarce, or a large number of people are vying for the same thing, it has a higher value.
3. The most recognized and liquid form of value is money. So, it is logical that if a post graduate course in radiology is so sought after by so many, it should cost 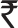 1.5 crore.

**The problem:** It is only a few who can recover the 1.5 crore in a reasonable breakeven time. So, the value given for a radiology seat is inflated. A person with the means to pay 1.5 crore is expected to estimate this: 15% returns from investing in a diagnostic facility plus the 1.5 crore is not a good deal.

**The real problem:** If MD Radiology “costs” that much, it means most post graduate aspirants cannot afford that – a value which not all those who have equal/more knowledge for a given seat could pay. So, most aspirants would not even apply for such a seat because they do not have the means. Thus, a person with less knowledge but more money would get the seat, and a higher academic qualification, probably sooner.

How often does ’Dr. 1.5 crore MD’ fail the final exam? How many do? Who would pay that magic sum for the next batch, if that happens? So, the very basis of our profession, reasoning 1 is being compromised.

Almost half of the post graduate seats are going under the hammer to the highest bidder. So, what happens to the larger number of have-nots who do not get a post graduation of choice, not because of the deficiency of requisite knowledge. Well, the system does offer an alternative: The National Board of Examinations (DNB), where one can pursue the speciality of choice, sans the 1.5 crore. But given the highest standards of education and a significantly lower pass percentage as compared to the MCI governed MD/MS system, with special reference to private medical colleges, only one party seems to benefit the most: “The Management” of private colleges demanding the 1.5 crore.

**Solution?** Ours is a country of inequalities. But this is more than just that. MD versus DNB is like the caste system denounced. For every point against, there will be a point proposed in favor. However, a good “litmus test” would be to implement a standardized post graduate examination for the entire country like the National Board of Examinations (DNB), in line with the analogous American Board. This would standardize post graduate medical education of our country in the true sense, including “1.5 crore” post graduation. To my mind, it seems to be a viable solution to bridge a gap that needs to be filled.

## References

[1] Jankharia B (2010). The business of radiology. Indian J Radiol Imaging.

